# Adherence to an enhanced recovery protocol in colorectal surgery before and during a structured implementation – a prospective cohort study from nine German hospitals

**DOI:** 10.1515/iss-2025-0004

**Published:** 2025-04-22

**Authors:** Wolfgang Schwenk, Christoph Strey, Sven Flemming, Monsserat Girona-Johannkämper, Wolfgang Wendt, Ibrahim Darwich, Hannes Neeff, Mark Banysch, Sandra Henn, Michael Scheruhn

**Affiliations:** Gesellschaft für Optimiertes PeriOperatives Management, GOPOM GmbH, Düsseldorf, Germany; Department of General and Visceral Surgery, DRK Krankenhaus Clementinenhaus, Hannover, Germany, (Chairman: PD Dr. med. C. Strey); Department of General, Visceral, Transplant, Vascular and Pediatric Surgery, University of Würzburg, Würzburg, Germany, (Chairman: Professor Dr. med. C. T. Germer); Department of Coloproctology, PROSELIS Stiftungsklinikum Prosper Hospital, Recklinghausen, Germany, (Chairman: Dr. med. E. Berg); Visceral Surgery/Proctology, Diakonissenkrankenhaus Dresden, Dresden, Germany, (Chairman: Dr. med. Th. Jacobi); Department of General and Visceral Surgery, St. Marien Krankenhaus Siegen, Siegen, Germany, (Chairman: Professor Dr. med. F. Willeke); Department of General and Visceral Surgery, University Hospital Freiburg, Freiburg, Germany, (Chairman: Professor Dr. med. S. Fichtner-Feigl); Surgical Clinic 1, St. Bernhard Hospital, Kamp-Lintfort, Germany, (Chairman: Professor Dr. med. G. Kaiser); Department of General- and Visceral Surgery, Rheinlandklinikum Lukaskrankenhaus, Neuss, Germany, (Chairman: Professor Dr. med. A. Ulrich); General- and Visceral Surgery, Krankenhaus Buchholz/Nordheide, Buchholz in der Nordheide, Germany, (Chairman: Dr. med. M. Scheruhn)

**Keywords:** enhanced recovery after surgery (ERAS), perioperative medicine, optimized perioperative management, learning curve

## Abstract

**Objectives:**

Enhanced recovery protocols (ERP) are considered as state of the art in perioperative management of elective colorectal resections. However, adoption of ERP in the clinical routine is not easy and a structured implementation process is recommended. The aim of the present data analysis was to evaluate the learning curve of the structured implementation of an enhanced recovery protocol in elective colorectal resections based on adherence to recommended ERP elements and clinical outcomes.

**Methods:**

During a 12-month structured implementation of an ERP in nine hospitals, perioperative management data were prospectively documented. Patients during the 3-month preparation phase were differentiated as a comparison group with traditional management from patients during the clinical ERP implementation. In addition, the 9-month ERP application phase was divided into three equal sections. Adherence to 23 recommended ER elements and to the pre-, intra- and postoperative elements was calculated.

**Results:**

One thousand one hundred fifty-three patients (48.3 % female) were included. Traditional perioperative treatments were applied in 313 patients (preERP), while 840 patients (249, 297 and 294 during months during the first [ERP3], second [ERP6] and last 3 months [ERP9] interval) were treated according to the enhanced recovery protocol. Overall preERP ERP-adherence was 52 (IQR: 48–57)% in 9 hospitals but increased to 87 (78–91)% at the end of ERP-implementation. Functional recovery after surgery increased from preERP to ERP9, and postoperative length of stay was reduced from 7 (6 – 8) to 5 (5 – 7) days. Major improvements in overall and preoperative ERP adherence were achieved during the first 3 months, while postoperative ERP adherence took 6–9 months to reach best results.

**Conclusions:**

Structured implementation of an enhanced recovery protocol resulted in high adherence to 23 ERP-elements within 12 months. Although major achievements occurred during the first 3 months of clinical ERP utilization, a total of 9 months are needed especially to improve postoperative ERP-adherence.

## Introduction

Although multimodal evidence-based concepts for perioperative management were developed more than 20 years ago [1] under terms such as FAST-TRACK [2], 3], enhanced recovery after surgery [4] or ERAS [5], their implementation in everyday clinical practice varies from country to country. Thus, enhanced recovery protocols are established as the standard of care in many clinics in Europe [6], [7], [8] and Canada [9]. In Germany, Austria [10] and the USA [11], the principles of enhanced recovery have been only recognized yet, but not completely established. There are various reasons why the establishment of ERP programs is so different encompassing structural and personal challenges, missing support from clinic management and lack of change management [12]. Furthermore, the exact duration of structured ERP implementation and the learning curve involved in such a process have not yet been discussed in detail in the literature. We therefore analyzed perioperative management data from nine German hospitals to describe changes in adherence to the ERP elements during a 12 month period of structured ERP implementation in more detail.

## Materials and methods

### Structured fast-track implementation

In all hospitals, structured implementation of an enhanced recovery protocol (ERP) was carried out according to the same scheme [13]. After the status quo of perioperative management had been determined an interdisciplinary enhanced recovery team consisting of nurses, surgeons and anesthetists was formed. Enhanced recovery nurses (ERP-nurses) were selected from the nursing staff to coordinate the interventions, care for the ERP patients, and collect clinically relevant perioperative management data [14]. The ERP team was trained in a workshop. Afterwards, the ERP and the necessary documents were created in a 3-month planning phase (preERP). Then the ERP protocol was used in the clinics from a fixed cut-off date. After 3 and 6 months of ERP use, workshops were held in the clinics in which the protocol was customized according to the special needs of each hospital. 12 months after the initial workshop, and 9 months after using the enhanced recovery protocol in the clinical routine, the ERP implementation was completed. At this time point, all clinics achieved the pre-defined targets for successful structured ERP implementation: ERP adherence >75 %, patient autonomy regained ≤5 days, postoperative length of stay <6 days, general complications <10 %.

### Patient population, inclusion and exclusion criteria

The data of all patients ≥18 years of age who were admitted for elective colorectal resection from the beginning of the structured implementation form the basis of the present data analysis. Patients who underwent multivisceral resections (i.e. small bowel, partial bladder resection, hysterectomy, partial vaginal resection or atypical resection of liver metastasis) were also included in the data collection. Patients who required emergency (<6 h after initial presentation) or urgent (6–24 h after initial presentation) surgery were excluded. Furthermore, patients scheduled for cytoreductive surgery with or without intraoperative hyperthermic chemotherapy, for pelvic exenteration including en bloc rectal resection with cystectomy (i.e., for recurrent rectal cancer) or simultaneous anatomical resection of liver metastasis were excluded. Furthermore, patients who did not consent to collection of their perioperative data were also excluded. Neither age, nor gender, indication for surgery, concomitant disease or risk factors were exclusion criteria. The structured implementation of the ERP is not a clinical study, but rather a quality assurance and improvement measure in everyday clinical practice. All perioperative and surgical procedures used are well established and represent standard procedures. Approval by an ethics committee was therefore not required. However, all patients signed an informed consent form for participation and data collection.

### Traditional treatment

During the planning of the fast-track treatment pathway (preERP), the established perioperative treatment for elective colorectal resections was not changed. During the initial workshop as well as in the subsequent video conferences, the treatment teams were encouraged not to change the existing perioperative treatment until the planning was completed.

### Enhanced recovery treatment

Optimized multimodal perioperative management was assessed according to international recommendations [15]. Following these recommendations, there were clear targets for 23 enhanced recovery elements (Table 1). To assess the progress of ERP implementation, the process was divided into 3 phases:

1. The start of the ERP application and the first customization workshop after 3 months (0–3 months; ERP3);
2. The first and second customization workshop (3–6 months; ERP6); and
3. The second adaptation workshop and the end of implementation (6–9 months; ERP9).

**Table 1:** Fast-track elements and adherence conditions.

Fast-track element	Adherence conditions
Nutritional status	Screening for malnutrition, preoperative nutrition therapy if necessary
Nicotine and alcohol abuse/abstinence	Screening for nicotine or alcohol abuse, 4-week abstinence before surgery if necessary
Patient education	Information and training on perioperative treatment, possibilities of participation, expectations, goals
Anemia	Anemia screening, specific therapy if necessary
Oral antibiotics	Oral antibiotics on the day before surgery
Metabolic conditioning	Oral administration of 12 % carbohydrate solution, 400 mL the day before surgery, 200 mL 2 h preoperatively
Bowel preparation	Refrain from mechanical bowel irrigation alone
Pharmacological premedication	No sedative medication
PONV prophylaxis	Risk-adapted combination prophylaxis
Antibiotic prophylaxis	Intravenous single-shot antibiotic administration up to 60 min before skin incision
Intraoperative opioids	No long-acting opioids
Regional analgesia	Thoracic peridural analgesia for open surgery, transversus abdominis plane block or spinal analgesia for MIS
Normothermia	Active heat supply through warm air blankets and heat mats
Gastric tube	No gastric tube or removal at the end of surgery
Drain	No drains
Minimal invasive surgery	Minimally invasive surgery if possible
Infusions	Balanced electrolyte solutions, maximum 3,000 mL on the whole day of surgery and no infusions from day 1 onwards
Nutrition	Protein-rich drinking solutions >200 mL on day of surgery and >400 mL on day 1 and >600 mL on day 2 and solid oral diet on day 1
Prophylaxis of GI-paralysis	Administration of magnesium or laxative from day 1
Enforced mobilization	Out of bed: ≥15 min on day of surgery and >4 h on day 1 and >6 h on day 2,. and >8 h on day 3
Urinary catheter	Colon resections: remove by morning of 1st day; rectal resections: remove by morning of 2nd day
Removal of epidural catheter	If present, remove on day 3
Follow-up	30-day follow-up by telephone or in person

### Postoperative complications

Adverse events that deviated from the unremarkable postoperative course until 30 days after discharge from hospital were considered complications and classified as general medical, local surgical and anesthesiologic complications. Any secondary procedure under anesthesia was documented as reoperation.

### Functional recovery

To describe functional recovery, the following were documented and analyzed: Interval between surgery and removal of the urinary catheter, tolerance of solid oral food, first bowel movement, regaining physical autonomy, and discharge from hospital. Autonomy was defined as the achievement of all the following goals: 1. reaching preoperative level of mobilisation (fully mobile, on crutches, wheelchair), 2. tolerance of oral food, passing stool, no i. v. fluids, 3. no nausea or vomiting, 4. getting dressed without help, 5. no i. v. analgesics.

### Data collection and analysis

Epidemiological data, disease, concomitant diseases, data on perioperative management, surgery and anesthesia, intra- and postoperative complications, and data on functional recovery as well as patients’ length of stay were prospectively collected in a pseudonymized manner. Data collection was carried out by ERP-nurses in the Interactive Fast-track Audit SysTem (INFAST^®^, GOPOM GmbH Düsseldorf) in the database management system Ninox^®^ (Ninox GmbH, Berlin). Adherence to the 23 fast-track elements ranged from 0 (none of the 23 elements utilized) to 100 (all 23 elements utilized) in each patient. Fast-track adherence was calculated for all patients as well as for each hospital. Statistical analysis of anonymized data was done in SAS-Studio^®^ (SAS Institute Inc., Cary NC, USA). Categorical data were tested for group differences using the chi2 test and Fisher’s exact test where appropriate. Continuous parameters are expressed as median (5th–95th percentiles) and group differences are analyzed with the *t*-test or Wilcoxon rank-sum test after testing for normal distribution.

## Results

The structured ERP implementation was done in the 9 clinics from 5/21/2021 to 10/31/2023. In these clinics, the implementation process lasted a median of 13.7 (25th–75th percentile: 13.5–14.1) months. Of this, 3.8 (3.5–4.1) months were spent on the pre-ERP phase, and 3.3 (3.1–3.5) months, 3.1 (2.8–3.4) months, and 3.1 (3.0–3.2) months on the ERP0-3, ERP3-6, and ERP6-9 phases. 313 patients underwent colorectal resection before ERP implementation (preERP) while 840 underwent surgery during clinical ERP implementation (ERP0–ERP9). The ERP3, ERP6 and ERP9 periods accounted for 249, 297, and 294 patients respectively.

### Patients and operative procedures

A total of 556 (48.3 %) women and 597 (51.7 %) men were treated, of whom 387 (33.6 %) were assigned to ASA classes III and IV. The average age was 66 (60–76) years and 644 (55.9 %) of the operations were performed to treat malignant diseases. Type of disease (benign vs. malign), type of resection (colonic vs. rectal) operative access (open vs. laparoscopic) are given in Table 2. The patients in the pre-ERP, ERP3, ERP6 and ERP9 groups did not differ in terms of gender, ASA classification, type and localization of the disease (Table 2). The age of the ERP3 patients was slightly higher than that of the other four groups (Table 2; p<0.03). 67.7 % of the preERP group underwent laparoscopic surgery, compared with 85.0 % of patients in the ERP9 group (Table 2; p<0.001).

**Table 2:** Epidemiological data and surgical procedures before ERP implementation (preERP) and during months 0–3 (ERP3), 3–6 (ERP6), 6–9 (ERP9) and overal of ERP implementation.

Patients	preERP		ERP3		ERP6		ERP9		p-Value^a^
	313		249		297		294		
	Median	25. – 75. %	Median	25. – 75. %	Median	25. – 75. %	Median	25. – 75. %	
Age	66	56–77	70	60–78	66	59–76	66	58–75	0.03
Sex									0.99
Female	150	47.9	120	48.2	144	48.5	142	48.3	
Male	163	52.1	129	51.8	153	51.5	152	51.7	
ASA-classification									0.23
I – II	212	67.7	152	61.0	200	67.3	202	68.7	
III – IV	101	32.3	97	39.0	97	32.7	92	31.3	
Type of disease									0.81
Benign	140	44.7	104	41.8	136	45.8	129	43.9	
Tumor	173	55.3	145	58.2	161	54.2	165	56.1	
Type of resection									0.78
Colonic	215	68.7	174	69.9	207	69.7	213	72.5	
Rectal	98	31.3	75	30.1	90	30.3	81	27.5	
Access									<0.001
Open	101	32.3	52	20.9	54	18.2	44	15.0	
MIS	212	67.7	197	79.1	243	81.8	250	85.0	

### Adherence to fast-track elements

**Figure 1:**
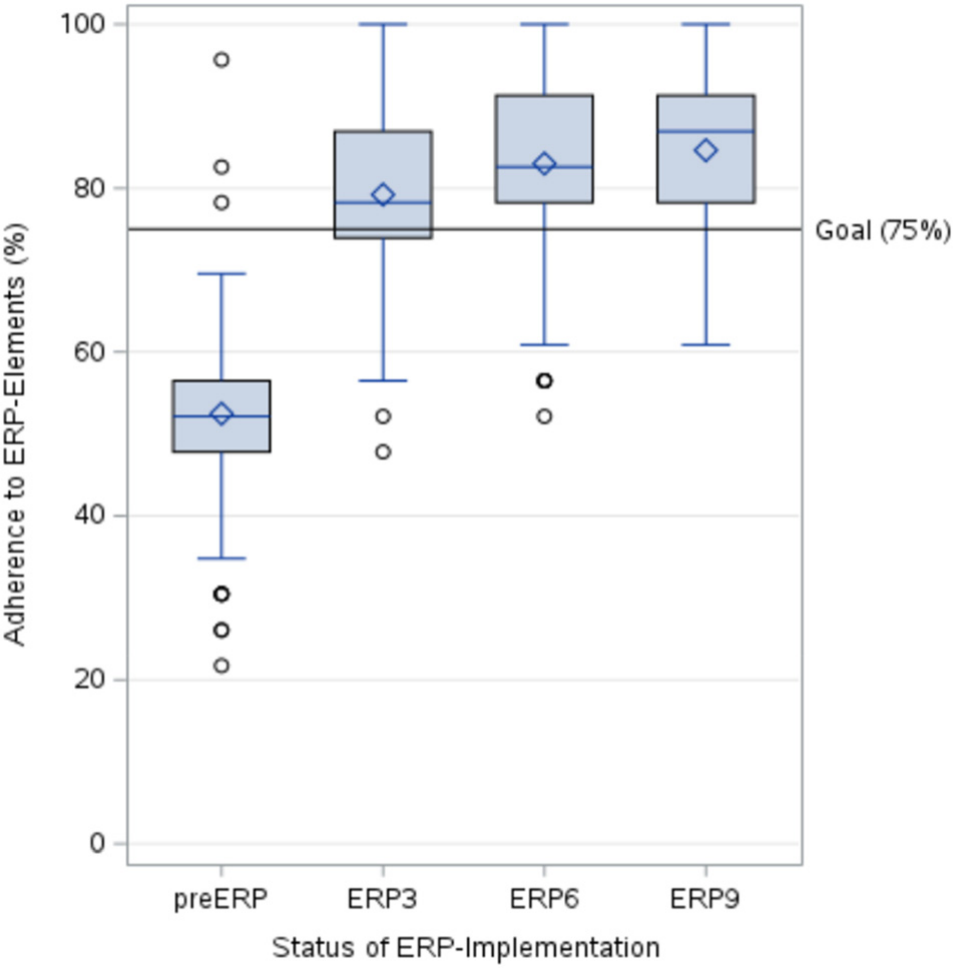
Adherences to ERP protocol before (preERP) and during months 0–3 (ERP3), 3–6 (ERP6) and 6–9 (ERP9) of ERP implementation.

**Figure 2:**
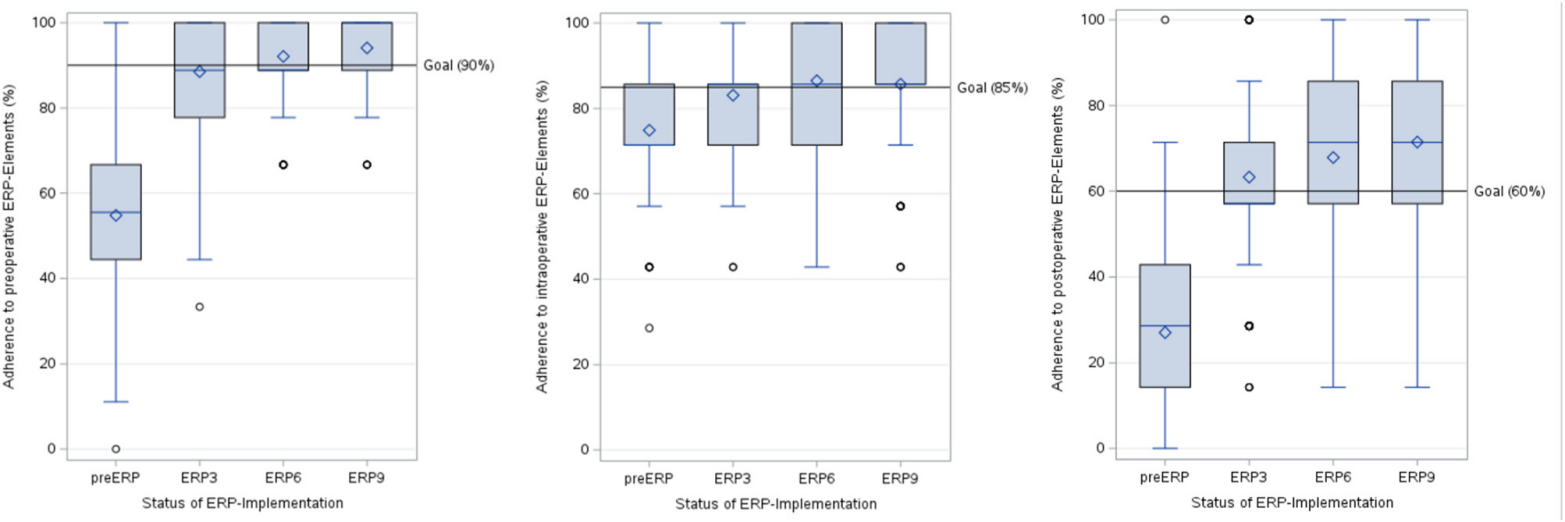
Adherences to pre-, intra-, and postoperative ERP protocol elements before (preERP) and during months 0–3 (ERP3), 3–6 (ERP6), and 6–9 (ERP9) of ERP implementation.

In the preERP phase, adherence to the 23 recommended ERP elements was 52 (48–57) % overall. Intraoperative adherence was higher at 71 (71–86) % than preoperative adherence at 56 (44–67) % and postoperative adherence was lowest at 29 (14–43) % (Table 3, Figure 1). ERP adherence increased most significantly in the first 3 months of clinical implementation, rising to 78 (74–87) %, with the largest adherence increase achieved in the preoperative phase (+33 %), while intraoperative adherence increased by 15 % and postoperative adherence by 26 % (Table 3, Figure 2). Over the next 3 months, overall adherence increased by a further 6 % (Figure 1). Preoperative and intraoperative adherence remained constant at 89% and 86 % respectively, while postoperative adherence increased by 14–71 % (Table 3, Figure 2). From ERP6 to ERP9, preoperative and intraoperative adherence remained stable at a very high level, while postoperative adherence increased by a further 4–87 % and thus accounted for most of the final overall adherence increase (Table 3, Figures 1 and 2).

**Table 3:** Adherence to ERP protocol before ERP implementation (preERP) and during months 0–3 (ERP3), 3–6 (ERP6), 6–9 (ERP9) and overall of ERP implementation.

	preERP		ERP3		ERP6		ERP9		p-Value^a^
	Median	25. – 75. %	Median	25. – 75. %	Median	25. – 75. %	Median	25. – 75. %	
Preoperative	56	44–67	89	78–100	80	89–100	100	89–100	<0.001
Intraoperative	71	71–86	86	71–86	86	71–100	86	86–100	<0.001
Postoperative	29	14–43	57	57–71	71	57–86	71	57–86	<0.001
Overall	52	48–57	78	74–87	83	78–91	87	78–91	<0.001

### Adherence to individual ERP elements

**Figure 3:**
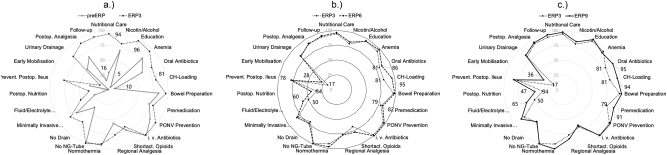
Changes in adherence to each element of the ERP protocol a.) before and during months 0–3 of ERP implementation; b.) during months 0–three and 3–6 of ERP implementation and c.) during months 0–3 and 6–9 of ERP implementation.

Figure 3a–c show a radar chart of adherence to the 23 ERP elements mentioned in the chronological perioperative course. PreERP, adherence only reached very high values for some elements of intraoperative management (e.g. iv. antibiotics and maintenance of normothermia). In contrast, postoperative adherence targets for early nutrition and early mobilization were not achieved in any patient (Figure 3a). In the first 3 months of ERP-use, there was a particularly strong increase in pre-admission and pre-operative adherence (e.g. nutritional screening and therapy, patient education, and metabolic conditioning with carbohydrate-rich drinks before surgery) and a few post-operative elements were also better implemented (Figure 3a). However, infusion therapy, rapid nutrition, and early mobilization continued to pose major challenges. Further improvements were observed for individual preoperative elements after 6 months (e.g. oral antibiotics, carbohydrate loading, and non-sedating premedication). Adherence to important elements was also increased postoperatively (e.g. infusion therapy, nutrition, and mobilization). Even at this time, only a few postoperative ERP elements achieved adherence of more than 75 % (Figure 3b). After a further 3 months, the use of the preoperative ERP elements has stabilized at a very high level (>85–90 %). During this period, further progress was made in the particularly demanding elements of infusion therapy, early nutrition, and rapid mobilization. Nevertheless, targets for mobilization and nutrition were still only achieved in less than half of the patients (Figure 3c).

### Morbidity and mortality

In 338 of the 1,153 patients, a complication occurred in the postoperative course up to 30 days after discharge (29.3 %). These were local surgical complications in 254 patients (22.0 %) and general medical complications in 130 cases (11.3 %). While there was no difference in the frequency of wound infections, anastomotic leaks or bleeding, the incidence of postoperative ileus (preventing oral feeding or requiring the insertion of a nasogastric tube) was higher in the ERP group (13.3 %) than in the pre-ERP group (4.5 %; p<0.01). Paralytic ileus was more frequently observed in the ERP9 than in the ERP6 or ERP3 period. Furthermore, there were no significant differences in the frequency of general complications. A total of 84 patients (8.9 %) underwent a second procedure due to complications, 95 (8.2 %) patients had to be readmitted to hospital within 30 days of discharge and seven patients (0.6 %) died after the elective procedure. There were no significant differences in the incidence of these adverse events between the preERP and ERP patients and no significant differences were found between the preERP, ERP3, ERP6 and ERP9 groups (Table 4).

**Table 4:** Postoperative complications (up to 30 days after discharge) before ERP implementation (preERP) and during months 0–3 (ERP3), months 3–6 (ERP6), and 6–9 (ERP9) months of ERP implementation.

	preERP		ERP3		ERP6		ERP9		p-Value^a^
	n	%	n	%	n	%	n	%	
Patients with complications	94	30.0	80	32.1	82	27.6	82	27.9	0.63
Patients with surgical complications	69	22.0	62	24.9	62	20.9	61	20.8	0.64
Surgical Site infection	41	13.1	20	8.0	28	9.4	22	7.5	0.09
Anastomotic leakage	20	6.4	15	6.0	8	2.9	13	4.4	0.14
Bleeding	7	2.2	7	2.8	7	2.4	10	3.4	0.80
Patients with general complications	42	13.4	31	12.5	31	10.4	26	8.9	0.29
Pulmonary	10	3.2	9	3.6	9	3.0	3	1.0	0.22
Urinary Tract related	8	2.6	7	2.8	9	3.0	8	2.7	0.99
Cardiac	9	2.9	5	2.0	13	4.4	3	1.0	0.07
Thromboembolic	4	1.3	1	0.4	1	0.3	2	0.7	0.49
Renal	1	0.3	2	0.8	0	0.0	1	0.3	0.47
Hepatic	0	0	1	0.4	2	0.7	0	0	0.30
Gastrointestinal bleeding	2	0.6	0	0	0	0	1	0.3	0.36
Neurologic	3	1.0	1	0.4	2	0.7	1	0.3	0.76
Delir	5	1.6	6	2.4	2	0.7	1	0.3	0.12
Sepsis	3	1.0	3	1.2	0	0.0	0	0.0	0.90
Patients undergoing re-operation	26	10.6	23	11.7	15	6.0	20	8.0	0.13
Patients readmitted to hospital	21	6.7	26	10.4	25	8.4	23	7.8	0.45

### Functional recovery and length of stay

The functional recovery of the patients was significantly faster in the ERP3, ERP6 and ERP9 groups than in the pre-ERP patients (Table 5). After just 3 months of ERP application patients showed a significant acceleration of postoperative recovery in all areas, and this continued in the ERP6 and ERP patients (Table 5).

**Table 5:** Postoperative functional recovery and length of stay before (preERP) and during months 0–3 (ERP3), 3–6 (ERP6), and 6–9 (ERP9) of ERP implementation.

	preERP		ERP3		ERP6		ERP9		p-Value
	Median	25. – 75. %	Median	25. – 75. %	Median	25. – 75. %	Median	25. – 75. %	
Interval from surgery to									
Removal of urinary cath. (P. o. day)	3	1–5	1	1–3	1	0–2	1	0–1	<0.001
Solid oral food, day	2	1–4	2	1–3	1	1–2	1	1–2	<0.001
First bowel movement (p. o. day)	2	1–3	2	1–3	2	1–2	2	1–2	<0.001
Regain Autonomy (p. o. day)	5	3–8	3	2–5	3	2–5	3	2–5	<0.001
Out of bed									
Day 1, hours	0	0–1	3	1–4.5	4	2–5	4.5	3.5–5.75	<0.001
Day 2. hours	0.25	0–3	4.75	2–6	6	4–7	6.25	5–8	<0.001
Day 3, hours	0.5	0–4	6	3.25–8	7.5	5–9	8,375	6–9.5	<0.001
Postoperative hospital stays, days	7	6–7	6	5–9	6	5–8	5	5–8	<0.001

The duration of patient mobilization from bed increased most significantly in the first 3 months of ERP application. However, a continuous increase in mobilization performance was shown after 6 and 9 months of ERP (Table 5, p<0.001). Due to the faster recovery, ERP patients could be discharged one day earlier (ERP3, ERP6) and finally two days earlier (ERP9) than preERP (p<0.001).

## Discussion

In nine German hospitals, the structured ERP implementation transformed traditional perioperative management with low adherence to internationally recommended enhanced recovery protocols (ERP) [15] into a modern evidence-based perioperative therapy with high ERP adherence. Postoperative recovery of patients was enhanced, and postoperative length of hospital stay was reduced. The greatest optimization of perioperative management was achieved through the first 3-months of clinical implementation resulting in an increase of ERP adherence from only 52 % with traditional therapy to 78 % with the ERP. This rapid initial increase was seen mainly in pre- and intra-operative ERP elements. 6 and 9 months after ERP implementation intra- and postoperative adherence further improved and an overall adherence above 80 % was achieved.

The joint definition of the treatment pathway by surgery, anesthesia, and nursing with the involvement of nutritionists, physiotherapists, and other professionals is the foundation on which the change from traditional to modern perioperative management rests [16]. However, as early as 2007, Maessen et al. described that an enhanced recovery protocol alone is not sufficient to ensure optimal adherence to the measures recommended therein. Rather, personnel, organizational, and structural measures must be considered [17], 18] to ensure that high adherence to the recommended measures is achieved. Ripolles-Melchor et al. [19], 20] and other authors [21], [22], [23] were able to convincingly demonstrate for colorectal resections that only an ERP adherence of 75 % or more leads to the effects of enhanced recovery described in the literature. To achieve high adherence there is agreement on the necessity of specialized enhanced recovery nurses to monitor and control the implementation of the treatment protocol in everyday clinical practice [14], 24], 25]. Interdisciplinary definition of the enhanced recovery protocol and strict monitoring of its implementation by the ERP-nurse, are the fundament for the massive increase in preoperative ERP-adherence from 56 % before to almost 100 % in the last 3 months of structured implementation (ERP9).

Even though the biggest jump in ERP implementation had occurred in the first 3 months, ERP adherence in the clinics continued to increase between the 3rd and 9th month. This ongoing improvement is based on the permanent monitoring of perioperative management using an audit system. Perioperative management data documented by the ERP-nurse in the audit system was fed back to the interdisciplinary treatment team via goal-defined dashboards showing the most important information. Specific measures to improve postoperative ERP-adherence included improved offers for patient mobilization, more precise integration of physiotherapy, and adapting nutrition to the needs of individual patients. By documenting the treatment, analyzing the results, providing feedback to the enhanced recovery team and jointly defining improvements, the further development of the ERP takes place within the framework of structured implementation in the sense of continuous quality management. It seems that in the last 3 months of the structured ERP implementation, little progress was made, so one could certainly discuss whether the implementation process could be shortened from 12 to 9 months. However, there was significant progress post-operative elements that are associated with ambitious goals, such as early nutrition and mobilization.

A critical observer reviewing the data presented could point out that even at the end of the structured ERP implementation, the postoperative hospitalization period was still relatively high at 5 days [26], 27]. In Germany, revenues are reduced by approximately €1,300 per day if patients are discharged any earlier then 4 days after colonic and 5 days after rectal surgery. Therefore, discharging patients any earlier will result in a loss of revenue of 10–12 % for the hospital. In contrast to data from randomized controlled trials [27], complication rate during the 9 months of ERP application was not significantly lower (21.1 %) compared to traditional care (25.9 %) (p>0.05), and general complications were as common during preERP (13.4 %) as during ERP application (10.5 %; p=0.17). However, when providing the ERP with an adherence of above 80 % to a further 733 patients after structured ERP implementation general morbidity was significantly reduced to 7.0 % (p<0.01) (data not shown). Furthermore, the change in perioperative management has not led to an increase in the frequency of wound healing disorders, anastomotic leakage or bleeding, nor has it changed the rate of reoperations in this population.

In summary, a 12-month transition from traditional to evidence-based perioperative care was successful in all participating hospitals. A clear learning curve was observed during the 9-month application of the hospital-specific enhanced recovery protocol. Although the changes were greatest in the first 3 months, further improvements were achieved in the following 6 months, resulting in a stable treatment concept in everyday clinical practice. A precisely formulated treatment protocol with clear treatment goals and supervision of patients and the treatment concept by specialized enhanced recovery nurses are a basic prerequisite for the success of the project, as is the use of an interactive audit system to monitor and continuously improve treatment.

## Appendix

Participating clinics, chief physicians or senior physicians of the surgical departments, FAST-TRACK coordinator, FAST-TRACK assistant (in alphabetical order of the clinics):

Diakonissenkrankenhaus Dresden,Visceral Surgery / Proctology: Dr. Th. Jacobi, Dr. W. Wendt, Fr. U. Meinhardt.

DRK Krankenhaus Clementinenhaus Hannover, General- and Visceral Surgery, DRK Krankenhaus Clementinenhaus Hannover: PD Dr. Ch. Strey, Hr. M. Dennin, Fr. T. Grönfeld.

PROSELIS Stiftungsklinikum Prosper Hospital, Recklinghausen, Department of Coloproctology, Fr. PD Dr. G. Böhm, Fr. Dr. M. Girona-Johannkämper, Fr. G. Özer.

Krankenhaus Buchholz/Nordheide, General- and Visceral Surgery: Dr. M. Scheruhn, Fr. B. Strauß, Fr. C. Kremer.

Rheinlandklinikum Lukaskrankenhaus, Neuss, Department of General- and Visceral Surgery: Prof. Dr. A. Ulrich, Fr. Dr. S. Henn, Fr. S. Brings.

St. Bernhard Hospital, Kamp-Lintfort, Surgical Clinic 1: Dr. M. Banysch, Fr. Dr. C. Bormann, Fr. K. Marx.

St. Marien Krankenhaus Siegen, Department of General and Visceral Surgery: Prof. Dr. F. Willeke, Dr. I Darwich, Fr. J. Heinemann.

University Hospital Freiburg, Department of General and Visceral Surgery,: Prof. Dr. S. Fichtner-Feigl, Prof. Dr. H. Neeff, Hr. B. Thoma.

University of Würzburg, Department of General, Visceral, Transplant, Vascular and Pediatric Surgery: Prof. Dr. C. T. Germer, PD Dr. S. Flemming, V. Bach, S. Böhm. G. Streahle.

## References

[1] Bardram L, Funch Jensen P, Jensen P, Crawford ME, Kehlet H (1995). Recovery after laparoscopic colonic surgery with epidural analgesia, and early oral nutrition and mobilisation. Lancet.

[2] Wilmore DW, Kehlet H (2001). Management of patients in fast track surgery. Br Med J.

[3] Basse L, Jacobsen DH, Billesbolle P, Kehlet H (2002). Colostomy closure after Hartmann’s procedure with fast-track rehabilitation. Dis Colon Rectum.

[4] Fearon KC, Ljungqvist O, Von Meyenfeldt M, Revhaug A, Dejong CH, Lassen K (2005). Enhanced recovery after surgery: a consensus review of clinical care for patients undergoing colonic resection. Clin Nutr.

[5] Varadhan KK, Neal KR, Dejong CH, Fearon KC, Ljungqvist O, Lobo DN (2010). The enhanced recovery after surgery (ERAS) pathway for patients undergoing major elective open colorectal surgery: a meta-analysis of randomized controlled trials. Clin Nutr.

[6] Bjerregaard F, Asklid D, Ljungqvist O, Elliot AH, Pekkari K, Gustafsson UO (2024). Risk factors for anastomotic leakage in colonic procedures within an ERAS-protocol. A retrospective cohort study from the Swedish part of the international ERAS-database. World J Surg.

[7] Gillissen F, Hoff C, Maessen JM, Winkens B, Teeuwen JH, von Meyenfeldt MF (2013). Structured synchronous implementation of an enhanced recovery program in elective colonic surgery in 33 hospitals in The Netherlands. World J Surg.

[8] Pache B, Martin D, Addor V, Demartines N, Hubner M (2021). Swiss validation of the enhanced recovery after surgery (ERAS) database. World J Surg.

[9] AlBalawi Z, Gramlich L, Nelson G, Senior P, Youngson E, McAlister FA (2018). The impact of the implementation of the enhanced recovery after surgery (ERAS((R))) program in an entire health system: a natural experiment in Alberta, Canada. World J Surg.

[10] Willis MA, Keller PS, Sommer N, Koch F, Ritz JP, Beyer K (2023). Adherence to fast track measures in colorectal surgery-a survey among German and Austrian surgeons. Int J Colorectal Dis.

[11] Shah TA, Knapp L, Cohen ME, Brethauer SA, Wick EC, Ko CY (2023). Truth of colorectal enhanced recovery programs: process measure compliance in 151 hospitals. J Am Coll Surg.

[12] Stone AB, Yuan CT, Rosen MA, Grant MC, Benishek LE, Hanahan E (2018). Barriers to and facilitators of implementing enhanced recovery pathways using an implementation framework: a systematic review. JAMA Surg.

[13] Schwenk W, Lang I, Huhn M (2021). Structured implementation of a fast-track program - how does it work?. Zentralbl Chir.

[14] Watson DJ (2018). Nurse coordinators and ERAS programs. Nurs Manag.

[15] Gustafsson UO, Scott MJ, Hubner M, Nygren J, Demartines N, Francis N (2019). Guidelines for perioperative care in elective colorectal surgery: enhanced recovery after surgery (ERAS^®^) society recommendations: 2018. World J Surg.

[16] Francis NK, Walker T, Carter F, Hübner M, Balfour A, Jakobsen DH (2018). Consensus on training and implementation of enhanced recovery after surgery: a Delphi study. World J Surg.

[17] Lam JY, Howlett A, McLuckie D, Stephen LM, Else SDN, Jones A (2021). Developing implementation strategies to adopt Enhanced Recovery after surgery (ERAS(R)) guidelines. BJS Open.

[18] Gramlich LM, Sheppard CE, Wasylak T, Gilmour LE, Ljungqvist O, Basualdo-Hammond C (2017). Implementation of Enhanced Recovery after Surgery: a strategy to transform surgical care across a health system. Implement Sci.

[19] Ripolles-Melchor J, Ramirez-Rodriguez JM, Casans-Frances R, Aldecoa C, Abad-Motos A, Logrono-Egea M (2019). Association between use of enhanced recovery after surgery protocol and postoperative complications in colorectal surgery: the postoperative outcomes within enhanced recovery after surgery protocol (POWER) study. JAMA Surg.

[20] Ripolles-Melchor J, Abad-Motos A, Cecconi M, Pearse R, Jaber S, Slim K (2022). Association between use of enhanced recovery after surgery protocols and postoperative complications in colorectal surgery in Europe: the EuroPOWER international observational study. J Clin Anesth.

[21] Maessen J, Dejong CH, Hausel J, Nygren J, Lassen K, Andersen J (2007). A protocol is not enough to implement an enhanced recovery programme for colorectal resection. Br J Surg.

[22] Burchard PR, Dave YA, Loria AP, Parikh NB, Pineda-Solis K, Ruffolo LI (2022). Early postoperative ERAS compliance predicts decreased length of stay and complications following liver resection. HPB (Oxford).

[23] Pędziwiatr M, Kisialeuski M, Wierdak M, Stanek M, Natkaniec M, Matłok M (2015). Early implementation of enhanced recovery after surgery (ERAS^®^) protocol – compliance improves outcomes: a prospective cohort study. Int J Surg.

[24] Pache B, Hübner M, Martin D, Addor V, Ljungqvist O, Demartines N (2021). Requirements for a successful enhanced recovery after surgery (ERAS) program: a multicenter international survey among ERAS nurses. Eur Surg.

[25] Wainwright TW (2020). The quality improvement challenge-how nurses and allied health professionals can solve the knowing-doing gap in enhanced recovery after surgery (ERAS). Medicina (Kaunas).

[26] Zheng V, Wee IJY, Abdullah HR, Tan S, Tan EKW, Seow-En I (2023). Same-day discharge (SDD) vs standard enhanced recovery after surgery (ERAS) protocols for major colorectal surgery: a systematic review. Int J Colorectal Dis.

[27] Greer NL, Gunnar WP, Dahm P, Lee AE, MacDonald R, Shaukat A (2018). Enhanced recovery protocols for adults undergoing colorectal surgery: a systematic review and meta-analysis. Dis Colon Rectum.

